# Biarticular muscles in light of template models, experiments and robotics: a review

**DOI:** 10.1098/rsif.2018.0413

**Published:** 2020-02-26

**Authors:** C. Schumacher, M. Sharbafi, A. Seyfarth, C. Rode

**Affiliations:** 1 Lauflabor Locomotion Laboratory, Centre for Cognitive Science, Institute of Sport Science, Technische Universität Darmstadt, Darmstadt, Germany; 2 Motion and Exercise Science, University of Stuttgart, Stuttgart, Germany

**Keywords:** biarticular muscles, leg morphology, locomotor subfunctions, biomechanical template models, legged robotics, assistive devices

## Abstract

Leg morphology is an important outcome of evolution. A remarkable morphological leg feature is the existence of biarticular muscles that span adjacent joints. Diverse studies from different fields of research suggest a less coherent understanding of the muscles’ functionality in cyclic, sagittal plane locomotion. We structured this review of biarticular muscle function by reflecting biomechanical template models, human experiments and robotic system designs. Within these approaches, we surveyed the contribution of biarticular muscles to the locomotor subfunctions (*stance*, *balance* and *swing*). While mono- and biarticular muscles do not show physiological differences, the reviewed studies provide evidence for complementary and locomotor subfunction-specific contributions of mono- and biarticular muscles. In *stance*, biarticular muscles coordinate joint movements, improve economy (e.g. by transferring energy) and secure the zig-zag configuration of the leg against joint overextension. These commonly known functions are extended by an explicit role of biarticular muscles in controlling the angular momentum for *balance* and *swing*. Human-like leg arrangement and intrinsic (compliant) properties of biarticular structures improve the controllability and energy efficiency of legged robots and assistive devices. Future interdisciplinary research on biarticular muscles should address their role for sensing and control as well as non-cyclic and/or non-sagittal motions, and non-static moment arms.

## 1. Introduction

Animals can easily perform a variety of movements. They coordinate their complex musculoskeletal system in a way that allows a simple description of the movement dynamics with template models [1]. For example, during running and walking, the dynamics of the human body—with all its segments and muscles—can be described with a leg-spring supporting the body mass [2–4]. These conceptual models suggest that the neural system controls the segmented leg in global coordinates like leg length and leg angle rather than individual joint angles [5–7]. The leg morphology and the muscle function certainly contribute to the simple behaviour of the complex leg [8].

In the sophisticated human leg, multiple joints need to be coordinated to generate global leg behaviour. Joint torques result from a superposition of torques generated by mono- and biarticular muscles. Monoarticular muscles act on one joint and thus tune single joint torques depending on neural stimulation. In contrast, biarticular muscles span two joints. Because the ratio of their muscle moment arms defines the ratio of biarticular torques, they embody specific coordination between joints. This built-in coordination likely has advantages as the leg morphology is the result of a long evolution [8,9]. However, current biomechanical analyses like inverse dynamics as well as typical robotic designs, focus on single joint dynamics (e.g. [10]) and thereby neglect inter-joint couplings and their potential in simplifying the realization of coordinated multi-segment motions [11]. The lacking understanding of biarticular muscle functions prevents exploitation of potential benefits of biarticular muscles, e.g. in the design of assistive devices. By taking a global perspective that focuses on the main limb behaviour, this review will fill the mentioned gap and identify specific contributions of biarticular muscles in the generation of cyclic human locomotion.

The specific functions of biarticular muscles have spurred the interest of researchers for centuries. Pioneers like DaVinci (1452–1519) exploited the ability of strings spanning multiple joints to transfer energy to move robots. Borelli (1608–1679) linked biarticular muscles to balance (drawing of a standing man with explicit biarticular muscle). The well-known *Lombard’s paradox* refers to the ability of a biarticular muscle to extend a joint that it anatomically flexes. The muscle extends the joint through the action of a co-contracted biarticular antagonist [11,12]. Biarticular muscles are believed to play an essential role in efficient [13] and robust [14] locomotion. However, despite the long history of research, the contribution of biarticular muscles for realizing locomotion is still not well understood.

In order to provide a new functional perspective on biarticular actuation, we split locomotion into the locomotor subfunctions *stance*, *balance* and *swing*. These subfunctions are derived from the global leg behaviour [15]. In contrast to single-joint analyses, this idea is based on simplified leg coordinates (leg length and leg orientation). We investigate the contribution of biarticular muscles to each of these subfunctions. To create a holistic overview, this article illuminates the two dimensions (i) locomotor subfunctions (stance, balance and swing) and (ii) different methodological approaches (theoretical concepts, experimental evidence and robotic applications) when reviewing biarticular muscle function.

In this review, we first define the framework of the locomotor subfunctions that is then used to examine biarticular muscle function in important conceptual models and experimental studies. Then, we review how hardware designs exploit biarticular structures in legged robots and assistive devices. We focus on cyclic tasks of human legged locomotion with a confined range of motion of lower-limb joints. Since the sagittal plane contains the majority of the leg’s range of motion and associated mechanical work generation in these tasks, other planes and associated biarticular functions (see e.g. [16]) are not considered. Finally, we integrate model predictions, human experiments and robotic applications into an overall picture of biarticular muscle function during cyclic locomotion and identify opportunities for future research.

## 2. Concepts and models of locomotion

### 2.1. Locomotor subfunctions

To build a common understanding and terminology, this section defines the framework of locomotor subfunctions that is used throughout this review.

Template models [1] can help to describe how the dynamics of the movement could be organized. These models have been used to resemble different features of legged locomotion at different levels of the human body (muscles, joints, segments). For instance, the behaviour of the leg during walking or running can be represented by the spring-loaded inverted pendulum, called SLIP model, that is universal for animals with different numbers of legs, humans and a variety of gaits [17–19]. This indicates a global organization of movement with functional requirements that are independent of the anatomical structure of the body. Agreeing with this, observations of animals and humans showed that individual joints are coordinated together to generate a desired behaviour of the limb or the whole body [5–7]. Legged locomotion can thus be considered as a composition of locomotor subfunctions that resembles the global organization of the corresponding limb to fulfil the functional requirement of the performed task. Previously, we proposed a set of three locomotor subfunctions, namely *stance*, *balance* and *swing* subfunction [20]:

— *Stance subfunction.* During ground contact, the stance leg exerts axial leg forces on the ground (at centre of pressure (CoP)) to counteract gravity and to redirect the movement of the body centre of mass (CoM).
— *Balance subfunction.* This subfunction represents a rotational body alignment to keep the upper body aligned vertically with respect to gravity.
— *Swing subfunction.* This subfunction controls the swing leg motion to prepare for the next ground contact. It comprises a rotational leg alignment adjusting the leg angle of attack and an axial leg length change, e.g. for foot clearance during forward swing.

Analysing separated locomotor subfunctions allows for the investigation of each individual subfunction at different levels [15] and their interaction [21]. These may involve experimental or computational approaches ranging from simplistic mechanical templates to complex neuromechanical models. With this approach, we aim to provide an integrative view on the upper body and lower limb functions which addresses the motion-dependent requirements of sensing, controlling and actuating all involved joints. Specifically, the approach will highlight the contribution of biarticular muscles by placing their features in a functional context for the coordination of multi-joint behaviours.

Locomotor subfunctions can be combined to create complex movements. This requires a certain degree of modularity to generate suitable combinations that coexist without disturbing or prohibiting each other.

In the current approach, some features of locomotion are simplified. Additional subfunctions could be identified in the future to extend or potentially revise the current composition of three locomotor subfunctions.

### 2.2. Biomechanical template models

**Figure 1.**
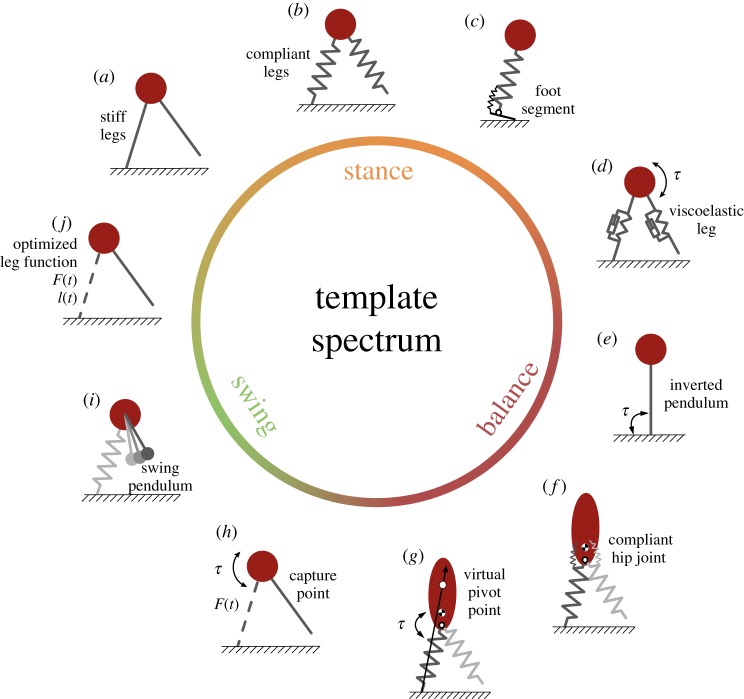
Selection of template models in the spectrum of locomotor subfunctions: (*a*) inverted pendulum (IP) model [22] and linear IP model [23], (*b*) spring-loaded inverted pendulum (SLIP) model [2–4,24], (*c*) SLIP model with foot (F-SLIP) [25], (*d*) hip-actuated-SLIP (Hip-SLIP) with damped leg [26], (*e*) ankle-actuated-IP model for standing [27], (*f*) SLIP with compliant hip and trunk [28,29], e.g. force-modulated compliant hip (FMCH) model [30], (*g*) virtual pivot point (VPP) model [31], (*h*) linearized hip-actuated-IP model with capture point [32], (*i*) swing pendulum model [33–35] and (*j*) optimized leg function [36]. Point masses neglect moment of inertia. Torques *τ* applied to the hip of a point mass are equivalent to models with upper bodies of infinite moment of inertia. Ellipsoid upper bodies have finite moment of inertia. Note that some references refer to slightly modified or extended versions of the shown models.

This section highlights fundamental mechanisms of bipedal locomotion to identify basic mechanical requirements of the locomotor subfunctions during cyclic motions like walking, running or hopping. A range of computational template models [1] that drastically reduce the complexity of the physical systems have been devised to understand fundamental mechanisms of bipedal locomotion. Most template models of locomotion contain, in a more or less abstract way, a combination of different locomotor subfunctions (see §2.1) that in an interplay enable cyclic locomotion. However, a single model often emphasizes a specific mechanism or function that particularly influences one or two locomotor subfunctions. According to these priorities, we arranged a number of used template models within the spectrum of locomotor subfunctions (figure 1). These template models have been used to analyse a variety of fundamental mechanisms of bipedal locomotion.

To investigate e.g. different gait patterns, gait stability or energy fluctuations, templates realized the specific subfunctions differently. For example, the stance subfunction has been described by rigid massless legs (figure 1*a*), compliant springs (figure 1*b*), viscoelastic elements (figure 1*d*) or time-dependent functions of length and force (figure 1*j*). The F-SLIP model (figure 1*c*) included a foot to investigate leg lengthening and the foot rollover.

In templates, the balance subfunction was realized by torques about the hip (figure 1*d*,*f*,*g*,*h*) or the ankle (figure 1*e*). Point mass models are reduced to investigating whole-body stability, i.e. CoM dynamics relative to the CoP. The balance subfunction for templates incorporating a trunk segment with finite moment of inertia (figure 1*f*,*g*) is more complex, because they simultaneously require upper body stability.

In most templates, the swing phase of the massless leg is reduced to defining an angle of attack (figure 1*b*). This neglects the leg dynamics. Other models consider motion dynamics (e.g. leg retraction [24,37], or capture point, figure 1*h*) to define the leg placement. Few investigations using templates have aimed to analyse the dynamics of the swing leg by representing the swing leg as a simple mechanical pendulum (figure 1*i*).

Templates are useful to understand locomotion on a global level and §4 will show their application in robotic systems (e.g. by virtual model control). However, in nature, legs are segmented and driven by mono- and biarticular muscles. Thus, the muscle’s mechanical function is strongly influenced by the leg architecture. This coupling also affects the neural coordination of the muscles. Since the mentioned template models abstracted this level of complexity, they are not suitable to study the neuromechanical realization of animal or human locomotion. To address questions concerning specific biarticular muscle functions or their neural control, the template models must be extended to segmented legs and (abstracted) muscles while preserving the derived concepts.

### 2.3. Biarticular structures in the segmented leg

**Figure 2.**
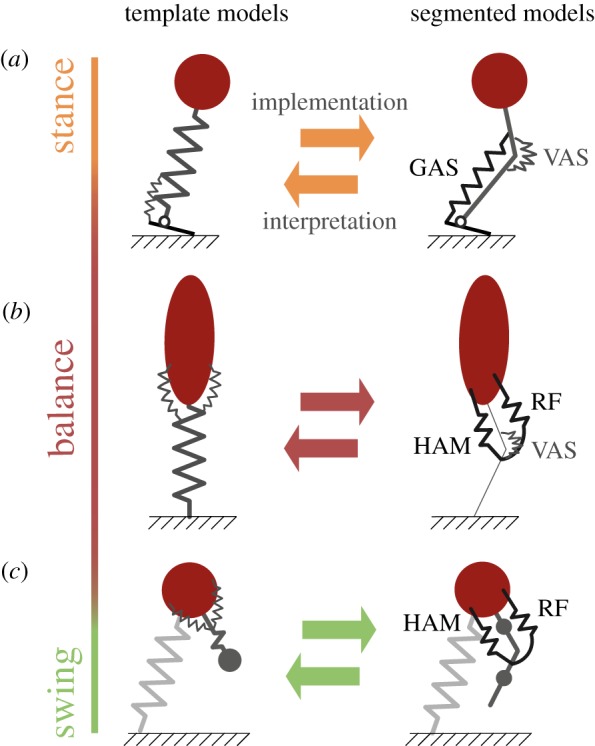
Biarticular structures bridge the gap between concepts (templates) and implementations (leg designs). Template models (with a telescopic leg structure) can be approximated with the use of biarticular muscles in the segmented leg (see text). Template models focus on fundamental mechanisms while segmented models can be used to study specific muscle functions. Equivalent template (left side) and segmented models (right side) for stance (*a*), balance (*b*) and swing subfunctions (*c*). Note that approximations are best for muscle moment arms ratios of 2 : 1 (hip to knee and ankle to knee) and equal segment lengths [38].

This section focuses on the relation between selected template and segmented models with abstracted muscles (figure 2) to examine how the fundamental mechanisms can be realized by biarticular structures. Compared to template-level analysis, segmented models resemble reality more closely. For example, compared to hip torques in a template model (e.g. figure 1*h*) which generate GRF perpendicular to the leg axis in the sagittal plane (rotary forces), monoarticular hip muscles in a segmented leg generate static forces aligned with the shank segment [39]. Thus, the direction of force generation with respect to leg axis depends on knee flexion angle. Nonetheless, the function of monoarticular hip muscles in the template model can be represented even in a segmented leg structure. Next to the contribution of two coordinated monoarticular muscles at the hip and the knee, this can be achieved by one biarticular thigh muscle with appropriate muscle moment arms resulting in substantial rotary forces [39]. For hip to knee muscle moment arm ratios of 2 : 1 and equal thigh and shank segment lengths, biarticular muscles generate GRF equal to hip torques in template models (figure 2*b*, [38]). With this leg architecture, the muscle–tendon length is proportional to the angle between the leg and the upper body [21,40].

Similarly, the gastrocnemius (GAS)^1^ muscle–tendon length is proportional to the angle between the leg and the ground for ankle-to-knee muscle moment arm ratios of 2 : 1 [39,40]. For flat foot contacts with the ground, GAS force generation mainly results in a horizontal force on the upper body [38]. This leg architecture was exploited to generate stable running with reduced control effort in a simulation model of a seven-link (trunk, thighs, shanks, feet) robot [40].

With a similar leg architecture, the segmented model in figure 2*c* predicted that biarticular muscles can be used to reconstruct the segment motions of the swing leg during human walking [41]. Additionally, biarticular springs (with an optimal hip-to-knee moment arm ratio of 3 : 1) allowed for a greater working range of walking speeds to a comparable subset of monoarticular muscles [14]. How these theoretical muscle moment arms relate to *in vivo* muscle moment arms will be discussed in §3.1.

These concepts suggest a relevant role of biarticular muscles to translate the fundamental mechanisms of legged locomotion—as predicted by the template models—into the segmented and complex leg physiology. By supporting the organism to benefit from these mechanisms (that reflect the underlying structure of locomotion) might be a crucial contribution of biarticular muscles. This theory demands further validation because simulation models and hardware demonstrators at this level of complexity are rare.

However, in theory, two monoarticular actuators can generate mechanically similar behaviours like a biarticular actuator. Through individual neural stimulation, two one-joint actuators can generate more flexible torque configurations [11]. This flexibility, however, comes at the cost of higher control effort and might be limited by neural or muscular constraints. In the case of steady-state cyclic locomotion, biarticular muscles might be sufficient to meet the simple and steady requirements of the locomotor subfunction: compliant axial leg function in *stance* (figure 1*b*,*c*,*d*), hip torques for upper-body *balance* (figure 1*f*,*g*,*h*) and passive *swing* leg dynamics (figure 1*i*). Also, muscle architectures and properties (§3.1) may determine if and when one strategy is preferred over the other.

## 3. Evidence of biarticular muscle function in bipedal locomotion

### 3.1. Muscle architecture and muscle properties

This section will discuss functional muscular adaptations like muscle architecture and selected properties. Even molecular muscle mechanics help to simplify control (e.g. [42–44]). A more exhaustive discussion of such specific muscle properties can be found in [38,45].

The muscle’s architecture and properties determine its ability to generate force and control the fibre length with respect to tendon stretch [46]. For example, short pinnate fascicles and long tendons are highly suitable for elastic recoil and economic force generation, while longer contractile fibres with short tendons allow for higher work generation and better controllability of the joint impedance, e.g. when facing perturbations [46,47].

In animals, biarticular muscles were found to cover a wide range of functions. Biarticular muscles damp impact-related oscillations in horses [48], allow for energy transfer in turkey, wallaby and goat distal hindlimbs or generate positive work in dog and goat forelimbs [49]. In contrast to the human leg, where biarticular muscles flex and extend adjacent joints, the biarticular *iliotibialis lateralis pars postacetabularis* in the guinea fowl extends both, hip and knee joints and undergoes a stretch–shortening cycle during stance [50,51]. Due to the diverse nature of biarticular muscle function in animal locomotion, we focus our analysis of muscle properties on the human leg.

**Figure 3.**
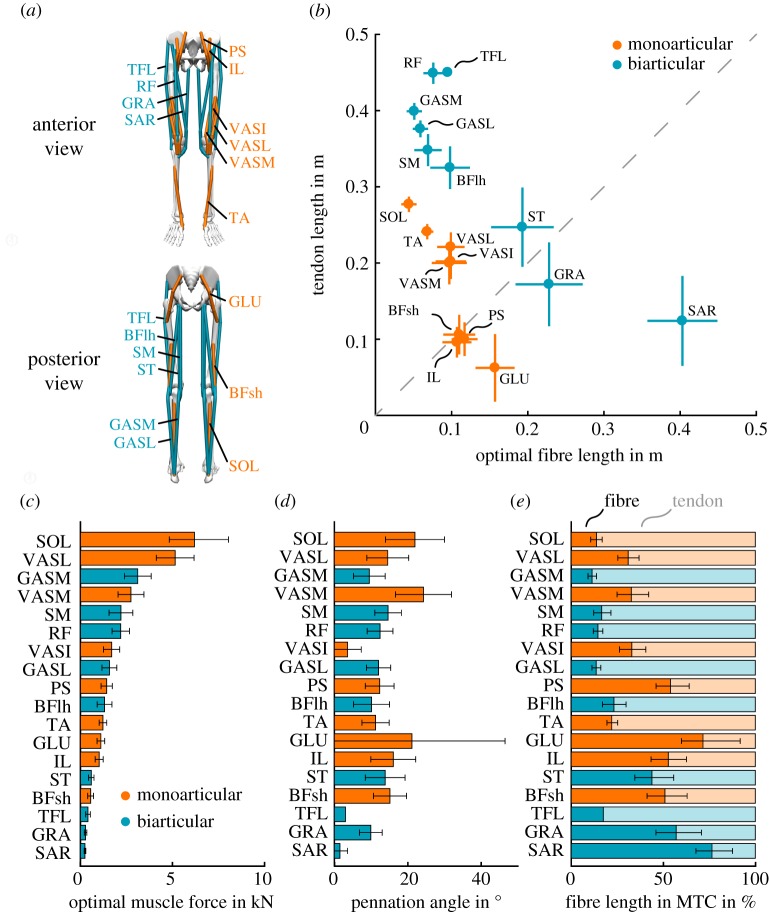
Overview of major mono- (orange) and biarticular (cyan) muscle architectures and properties. Figures were generated based on data from Rajagopal *et al.* [52]. (*a*) Arrangement of anterior (top panel) and posterior (bottom panel) muscles groups. (*b*) Mean and standard deviation (SD) of tendon and muscle fibre lengths. (*c*) Mean and SD force capacity of muscles. (*d*) Mean and SD pennation angles of muscles fibres. (*e*) Mean and SD distribution of muscles fibres and tendon lengths in the MTC. Muscle abbreviations: soleus (SOL), vastus lateralis (VASL), gastrocnemius medial head (GASM), vastus medialis (VASM), semimembranosus (SM), rectus femoris (RF), vastus intermedius (VASI), gastrocnemius lateral head (GASL), psoas major (PS), biceps femoris long head (BFlh), tibialis anterior (TA), gluteus maximus (GLU), iliacus (IL), semitendinosus (ST), biceps femoris short head (BFsh), tensor fasciae latae (TFL), gracilis (GRA) and sartorius (SAR). Note that for TFL no variances of optimal fibre length, tendon slack length and pennation angle were reported in Rajagopal *et al.* [52].

To check for specialized biarticular muscle designs, we visualized data of human leg muscles (figure 3*a*) stemming from Rajagopal *et al.* [52]. Figure 3*b* shows that the muscle tendon complex (MTC) of biarticular muscles is generally longer than that of monoarticular muscles. This result is explained by the definition of bi- or multiarticular muscles as they span more than one joint or segment. The additional length of the MTC must thereby stem from longer tendinous structures and/or longer contractile fibres.

The contraction dynamics and function of muscles depends on their fibre–tendon length ratio [53,54]. Major muscles undergoing stretch–shortening cycles during walking, e.g. during ankle push-off (stance subfunction, e.g. SOL, VAS, GAS) or swing leg acceleration (swing subfunction, e.g. RF, ST, BFlh), mainly use longer tendons (figure 3*b*), supporting principles of energy storage and return to reduce the energy requirement of walking [55–59]. In addition, due to the serial arrangement of fibres and tendons, these muscles must be able to make use of the tendon’s recoil [46]. In accordance with this requirement, these muscles show high force capabilities (figure 3*c*) and highly pennated muscle fibres (figure 3*d*) generating high output forces to load the relatively long tendons (figure 3*e*).

Muscles with longer fibres relative to tendon length (figure 3*e*: SAR, GRA) were associated with leg rotation in the transversal plane and thus steering [16] and might relate to leg joint stability. Their long contractile fibres and short tendons allow a more direct control of the muscular impedance [46]. Typically, steering does not involve powerful stretch–shortening cycles of these muscles. This is reflected in their limited output force, as found by a small force-generating capacity (figure 3*c*) and small pennation angles (figure 3*d*).

These results show that mono- and biarticular muscles share similar properties (figure 3; [11]). The muscle’s specific characteristics may be more a result of a continuous adaptation to the organism’s lifestyle and its environment [60,61].

**Figure 4.**
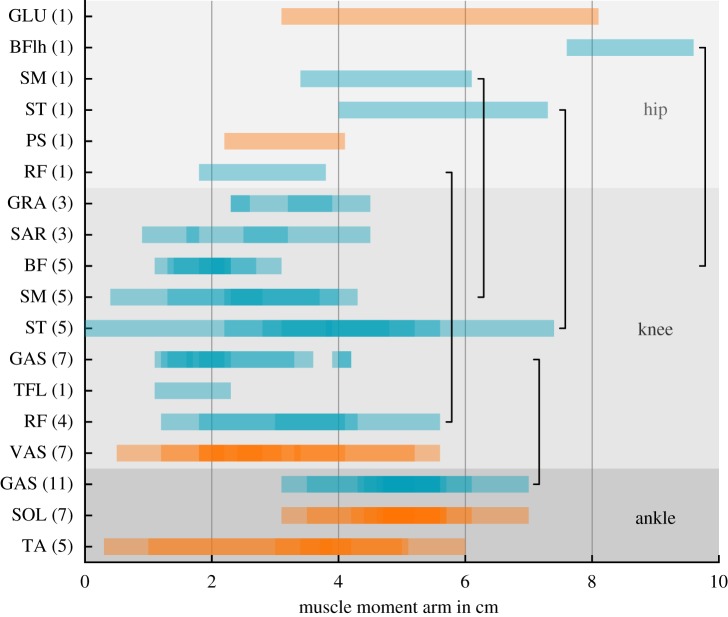
Range of sagittal muscle moment arms for human mono- (orange) and biarticular (cyan) leg muscles. Darker colour bars shows overlaying results from multiple studies. Note that working ranges and methods of assessment vary between studies. Numbers in parentheses denote the number of studies that are shown for that muscle. Black lines connect both joint moment arms of the same biarticular muscle. Data for visualization stems from: Németh & Ohlsén [64], Visser *et al.* [65], Arnold *et al.* [66] (hip), Wretenberg *et al.* [67], Buford *et al.* [68], Spoor & Van Leeuwen [69], Maganaris *et al.* [70], Herzog & Read [71], Visser *et al.* [65], Arnold *et al.* [66] (knee) and Maganaris *et al.* [72,73], Maganaris [74–76], Fath *et al.* [77], Karamanidis *et al.* [78], Hashizume *et al.* [79], Rugg *et al.* [80], Klein *et al.* [81], Sheehan [82] (ankle). Used muscle abbreviations are gluteus maximus (GLU), biceps femoris long head (BFlh), semimembranosus (SM), semitendinosus (ST), psoas major (PS), rectus femoris (RF), gracilis (GRA), sartorius (SAR), biceps femoris long head (BFlh), gastrocnemius (GAS), tensor fasciae latae (TFL), vastus (VAS), soleus (SOL) and tibialis anterior (TA). BF denotes knee moment arms of biceps femoris long head (BFlh) and biceps femoris short head (BFsh). For HAM muscle group and GAS, also see Cleather *et al.* [62].

Moment arms of biarticular muscles are important for understanding their functional contribution to generate torques at the joints they span [41,62,63]. As discussed in the previous section, appropriate biarticular moment arms translated fundamental mechanisms of template models to more elaborate leg designs. To check if these assumptions reflect the human physiology, we visualized data from *in vivo* and *in vitro* studies that investigated human muscle moment arms in the sagittal plane. Since muscle moment arms depend on joint angles, results vary due to a wide range of studied joint angles (figure 4). The biarticular GAS moment arm at the ankle was found to be in range of 3 to 7 cm while its knee arm was in range of 1 to 4 cm. For biarticular thigh muscles, hip moment arms seem to be greater than knee moment arms (figure 4 and Cleather *et al.* [62]). The results presented here seem to roughly agree with previously postulated [10] moment arm ratios of GAS (ankle to knee ratio: 2 : 1), HAM (hip to knee ratio: 2 : 1) and RF (hip to knee ratio: 4 : 3).

Only a limited number of studies quantified the hip moment arms. In addition, moment arms can undergo substantial changes, e.g. during a normal stride, as shown by a hindlimb model of the rat [63]. Even though such changes might be less pronounced for humans—as they use more extended leg configurations during locomotion—the provided overview should only be used as a guideline. In order to draw reliable conclusions further studies are needed.

### 3.2. Biarticular muscles in human locomotion

In this section, we will review evidence from experimental studies that have investigated the role of biarticular muscles in human locomotion. We categorize the existing works by locomotor subfunctions and their task- and function-specific context.

#### 3.2.1. Stance

In animals and humans, withstanding or overcoming gravitation is a major requirement for locomotion. During ground contact, leg joints (hip, knee and ankle) are often synchronized such that they undergo a flexion/extension when the leg is shortened/extended, respectively [7,83,84]. Due to the zig-zag configuration of the leg, biarticular leg muscles are then simultaneously pulled at one joint and released at the other joint. The length of the biarticular muscle tendon complex has been found to remain almost constant for both extremes of the full leg length range of motion [83]. Other studies have found that the MTC of the GAS does stretch during the stance phase of walking, but that the stretch appeared in the tendon, allowing the fascicles to operate almost isometrically [85–88]. This held even over a range of ground inclines or speeds, when higher work generation is required [89]. Such isometric contraction of the biarticular fascicles is beneficial for multiple reasons. An isometric contraction delivers a higher force compared to a shortening contraction due to the force–velocity relationship of muscle force production [90]. Moreover, the tensed biarticular MTC is able to transmit forces to neighbouring joints, while generating almost no mechanical work (product of force and contraction velocity). This is advantageous because such a close to isometric contraction (almost zero velocity) requires less metabolic cost compared to quicker contractions [91]. Two monoarticular muscles substituting one biarticular muscle would have to be activated and undergo shortening/lengthening contractions, respectively, to achieve a similar mechanical outcome at a higher cost [92–94].

If a biarticular muscle is co-contracted with an adjacent monoarticular muscle, the monoarticular muscle can act on a joint that it does not span. For example, a hip extension (e.g. by GLU) can be transferred via a ligamentous action [83] of the RF to a knee extension. By such coupling more powerful muscle groups (with greater muscle volume) can contribute to the net torque of an adjacent joint [95–97]. Researchers concluded that such joint coupling is an effective strategy to reduce distal mass of the legs and minimize the mechanical delay of the system in response to neural commands [92,95–98].

During push-off in jumping, the major leg joints extend in a temporal sequence from the hip to the ankle enabling an energy flow from proximal to distal joints [95,98]. It was found that the biarticular joint coupling enabled a more efficient execution of the push-off [98]. Prilutsky & Zatsiorsky [97] also showed that such energy flow can be effective vice versa (from distal to proximal joints) to dissipate energy in the powerful proximal muscles, e.g. during landing or load response.

During quick knee extensions at the end of stance in hopping, the biarticular GAS transformed rotational kinetic energy of thigh and shank to a translational push-off motion [98,99]. This not only improved the legs push-off performance but also prevented knee overextension [98,99]. During hopping, such energy transfer (from knee to ankle) could contribute up to 25% to the peak power output at the ankle [100,101].

#### 3.2.2. Balance

While the term balance usually refers to the task of maintaining stability of the whole body, here, we consider the postural control of the upper body to focus on the involvement of biarticular thigh muscles for generating stabilizing hip torques [27,102].

The ability of biarticular muscles to mainly contribute to rotary leg forces [39] can be especially useful for controlling angular momentum and thus postural balance. In this context, a study investigated the reaction of subjects who stood on one leg to maintain their posture while being exerted to external (anterior and posterior) forces on the unloaded leg [38]. In response to the introduced joint torques, subjects dominantly (and consistently) recruited biarticular thigh muscles in both legs, while EMG activity in monoarticular muscles changed inconsistently [38].

Further experiments have studied the relation of biarticular muscle function and appropriate combinations of hip and knee joint torques (and associated GRF adaptations). Doorenbosch & van Ingen Schenau [103] reported high correlations (0.935 ± 0.027 s.d.) between the isometric muscular activity of RF and HAM muscles and the net joint torque of hip and knee. For a desired combination of hip flexion and knee extension torque, increasing RF and decreasing HAM activation was observed. Both antagonistic biarticular thigh muscles were recruited in a reciprocal way, depending on the torque requirements of the tasks [103–106]. Similar patterns for GRF manipulations were also observed in cycling [107] or load lifting tasks [108,109].

To also shed more light on reactive control strategies to unexpected and immediate perturbations, we recently applied impulse-like pitch perturbations to the upper-body during standing [110]. In line with the studies above, biarticular thigh muscles had the strongest increase in muscular activity of all measured muscles (monoarticular hip muscles showed only moderate to no reactions). These results provide further evidence that RF and HAM actively control the required net hip to knee torques coordinating the posture of the upper body [110].

#### 3.2.3. Swing

In bipedal locomotion, the swing leg performs a forward motion while being unloaded. The swing phase requires suitable swing leg length and orientation trajectories to achieve ground clearance (avoiding obstacles) and a proper foot placement for the next stance phase. Since only inertia is to be overcome, required joint torques are rather small compared to the stance subfunction [111]. However, an important requirement lies in the proper coordination and synchronization of different joints.

RF and HAM experience stretch–shortening cycles facilitating energy store-and-release mechanisms during walking, running and sprinting [59,112]. In late stance, RF length increased (also loading the tendon) due to hip extension. Together with a concentric contraction, this energy was released and helped to initialize the forward swing of the leg. For HAM, the stretch–shortening cycle appeared during the forward swing and subsequent retraction of the leg. While the elastic energy storage serves to improve the horizontal propulsion, previously mentioned template models revealed benefits of such leg retraction strategy (here by HAM) on running stability [37,113]. Both biarticular thigh muscles exchanged energy between stance and swing phases [59]. However, only data from two subjects were assessed, revealing the demand for further experimental support of these mechanisms.

Prilutsky *et al.* [108] investigated the role of biarticular thigh muscles during the swing phase of walking and running at different speeds. Phase-specific contributions of RF and HAM for specific hip and knee torque combinations were found. Similar to balancing the upper body (see §3.2.2), RF and HAM of the swing leg showed reciprocal EMG patterns in line with the net hip to knee torque requirement. Muscle activation of the RF was significantly higher during the early half of the swing phase, when hip flexion and knee extension torque occur simultaneously, compared to the second half. For HAM, the opposite was reported. These patterns occurred in both walking and running gaits. Authors also found high correlations for the EMG difference of RF and HAM with the net hip to knee torque (between 0.923 and 0.959 for different speeds). However, since only four subjects participated in the study, and a total of only three swing phases each were used for the analysis, more quantitative data should confirm these results.

### 3.3. Biarticular sensors

In addition to generating appropriate joint torques throughout the segmental chain [97,108,114], biarticular muscles might also play an important role for sensing limb posture in global coordinates, e.g. limb orientation and length [7,115–118]. Potential implementations could involve force feedback, e.g. by the respective Golgi tendon organs [108,119] or by cutaneous receptors in the foot sole, sensing the leg force [21,30,108,120]. In this context, Lacquaniti & Soechting [121,122] and Soechting & Lacquaniti [123] found that, following torque perturbations at the arm, the effective net torque of the elbow and shoulder was a better predictor for the observed muscle responses than single joint angular velocities (and individual stretch reflexes). Similar results were also found in the leg [103,104,106]. As the length of HAM and RF remains almost constant when the leg length shortens or extends [83,84], these muscles predominantly undergo a change in length when e.g. the trunk orientation changes with respect to the leg orientation (see discussion in §2.3). Thus, length feedback pathways of biarticular muscles could sense the orientation of the limb axis directly. By this, biarticular muscles provide a simple solution of postural proprioception, complementing vestibular sensation and other sources in postural equilibrium tasks.

The sensing of leg length and orientation [7,115,116] by biarticular muscles could simplify control and coordination of joints [40,117,124]. In this context, a parallel and independent control of axial and perpendicular leg forces by mono- and biarticular have been suggested from studying the perturbation response of standing cats [114,125]. However, more research is required to identify specific biarticular reflex pathways and the corresponding involvement in generating appropriate movements, also in the context of muscle synergies [126,127].

## 4. Applications in robotic devices

In this section, we review the application of biarticular elements (e.g. actuators or springs) in the design of legged robotic systems. For this, we will present how compliant biarticular structures were used to improve the controllability of these systems, before we review different hardware designs and control concepts in the light of the locomotor subfunctions.

### 4.1. Control embodiment via compliance and biarticular mechanism

The morphology and biomechanics of humans and animals have great impact on locomotion control [128]. This was formulated in the *control embodiment*^2^ concept, in which the mechanical structure is considered to be an important contributor for generating appropriate movements and solving control challenges [128].

In the context of locomotion, robotic systems used biarticular structures with compliant properties of muscles or tendons [124,130–135]. Such designs are inspired by biological bodies [136,137]. Both of these qualities, biarticular arrangement and inherent compliant properties, can be considered tools for control embodiment. They can improve the controllability of the robot, enable energy management and improve robustness against the uncertainty of the environment, e.g. changing terrains [138,139]. Additionally, passive mechanical structures instantaneously interact with the environment and can thus respond to external perturbation without a control delay. Examples of these benefits can be found in bipedal and quadruped robots, where biarticular springs helped to generate stable gaits even when a simple open loop control (without sensory feedback) was used [132,134,140,141].

As discussed in §§2.3 and 3.3, the length of the biarticular spring—given appropriate moment arm ratios—corresponds to rotational changes of the whole leg with respect to the adjacent segment. For instance, the length of a biarticular thigh spring (similar to HAM or RF) can be proportional to the angle between the leg and the upper body [21,40]. By this, a biarticular spring could be used to directly react to perturbations on the upper-body posture. The results of Schumacher *et al.* [110] support the beneficial contribution of biarticular structures to recover from upper-body perturbations in human-like leg designs. In another example of a simple swing leg model, the (rest) length of biarticular thigh springs was found to linearly correlate with the target swing leg angle [41]. However, in all these applications, biarticular moment arm ratios are an important factor determining the functional contribution of the biarticular actuator or spring, as shown in the *CARL* robot [142]. Proper design of moment arm ratios and spring properties (e.g. spring stiffness or rest length) can thus be used as design parameters to determine the desired limb behaviour and incorporate a bioinspired control embodiment strategy in robotic systems [40,117,124,142,143].

### 4.2. Biarticular structures in legged robots

**Figure 5.**
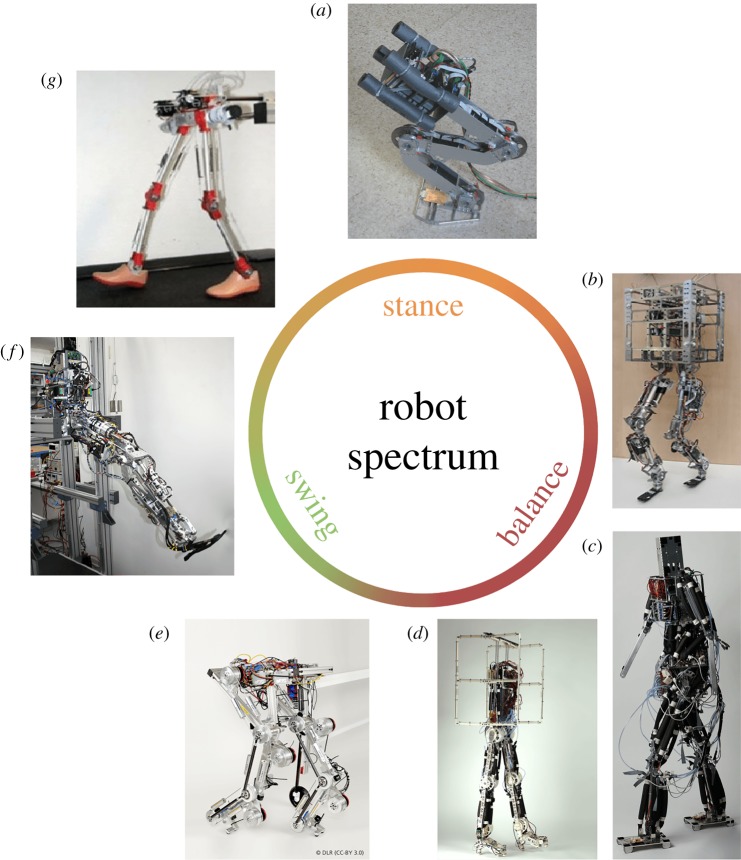
Selection of simple and complex legged robots using biarticular actuation in the spectrum of locomotor subfunctions. (*a*) Biarticular legged robot [144,145], (*b*) *BioBiped* [117], (*c*) *Pneumat-BS* [134], (*d*) *Pneumat-BB* [146], (*e*) *C-Runner humanoid running machine* [124,147], (*f*) *CARL* robot [148] and (*g*) *Jena Walker II* [149]. Note that some of the shown robots also cover multiple subfunctions, but were arranged according to the main functional contribution of biarticular actuation. Photos in the courtesy of: Jan Babič (*a*), Koh Hosoda (*c* and *d*), Patrick Vonwirth (*f*) as well as under CC BY 3.0 from DLR (*e*) and [117] (*b* and *g*).

Several robotic systems investigated the effect of biarticular actuation (figure 5) to counteract gravity (stance subfunction) in dynamic motions by evaluating the motion performance, e.g. the hopping height. In the study of Hosoda *et al.* [131], biarticular pneumatic artificial muscles (PAM) were used to transmit joint torques along multiple segments and coordinate multiple joints in vertical jumping. Further, a hopping robot [144,145] was used to test the results of a computational study that predicted improvements in hopping performance due to GAS muscle energy transfer from knee to ankle [101]. The hopping robot confirmed that hopping height increased due to an additional energy-storage and release in the added GAS as well as improved energy transfer from proximal to distal joints. With an appropriate timing and magnitude of GAS actuation, hopping height increased by 18% [145]. For *CARL*, energy transfer of biarticular actuators improved the overall jumping efficiency of the robotic leg [150]. Similar effects were also reported in the *BioBiped* robot and free falling experiments [117,151]. In another study, a three segmented mono-pedal hopping robot with a point foot used electromagnetic linear actuators to mimic compliant mono- and biarticular muscles [139]. In this system, biarticular thigh muscles tuned the *stiffness ellipse*^3^ at the foot during stance and consequently, controlled the motion direction of the robot in the flight phase.

The mechanism of a proximal-to-distal energy transfer with biarticular muscles (see §3) was further tested in a human-sized robotic leg that used all major monoarticular (except the knee flexor) and biarticular RF and GAS actuators to perform squatting movements [152]. It was found that, if force generation of GAS precedes SOL contribution, the total ankle power increased, compared to simultaneous force generation.

Biarticular actuation was used to improve the postural balance function in robots, e.g. by PAM in the *Pneumat-BS* [134] and the *Pneumat-BB* [146] or by serial elastic actuator (SEA) in the *BioBiped* [117]. In standing and squatting experiments of the *BioBiped3* robot, the cross-talk between axial and perpendicular terms of GRF was reduced when hip-to-knee moment arm ratios approached values of 2 : 1 [117]. By this, VAS and biarticular thigh muscles could respectively control GRF magnitude and direction with minimum interference. This agrees well with results from simulations and experiments (see §§2 and 3 for details) and further suggests that the two subfunctions of stance and balance could be assigned to different muscle groups.

Inspired by simulation results [41], a simulation model of the *BioBiped* robot used biarticular thigh actuators to generate an appropriate leg swing motion for forward hopping [117,153]. For this, a pre-tension of the elastic element in the SEA (during late stance) resulted in a passive response of the spring realizing the leg swing motion [117]. Further, simulation results of a running robot [40] and successful implementation of a simple controller exploiting biarticular SEA in a humanoid robot achieving dynamic walking [124] indicate that for an appropriate design of biarticular actuators, balance and swing subfunctions could be decoupled from the *stance* control.

By such muscle-specific task allocation, control approaches could be simplified to setting properties of compliant elements (e.g. spring stiffness or rest length) to a specific value for each gait condition. An example of this can be found in the *Jena Walker* robot [132,140]. Combining oscillatory feed-forward actuation at the hip for propulsion (push-off and leg swing), a monoarticular spring (like TA) and multiple biarticular springs (similar to RF, HAM and GAS) generated stable walking and running patterns. By this, stance and swing subfunctions were nicely coordinated using passive biarticular springs while the TA spring was mainly used for foot clearance [132,140]. To further coordinate or synchronize different subfunctions, e.g. when facing perturbations, sensory feedback pathways can be used. In that context, leg force feedback could coordinate subfunctions of stance (VAS) and balance (RF and HAM) and improve the robustness against perturbations in simulation [21].

### 4.3. Biarticular structures in assistive devices

Recently,^4^ biarticular structures were used in the design of assistive devices for impaired [154,155] and non-impaired people [156,157].

In walking experiments of unilateral amputees, Eilenberg *et al.* [155] emulated the healthy human GAS behaviour by combining a powered ankle–foot prosthesis and a robotic knee orthosis. In this study, neuromuscular models of matched non-amputees were used to model the lower limb biarticular muscle. It was hypothesized that by biarticular coupling with an artificial GAS, the active push-off of the prosthesis could be used to reduce the work in the affected-side hip and knee during leg swing initiation. Compared to a monoarticular operation (without knee orthosis contribution), reductions in biological knee flexion moment impulse of the affected-side as well as reduced positive work of the hip during late-stance knee flexion were found. This resulted in decreased metabolic power during walking in some subjects (four out of six), although non-significant over all subjects [155].

For assisting non-impaired people, the application of biarticular structures in *exosuits* became more popular, due to the soft and flexible design of muscle-like, tension-based actuation principles [156,157]. Since actuators can also span multiple joints in these systems, simulation models were used to identify an optimal actuator arrangement in the *exosuit*. Sharbafi *et al.* [158] extended the neuromuscular walking model of Geyer & Herr [159] by a virtual HAM-like actuator with FMCH-based control. While parameters of the original simulation model remained unchanged, simulation results predicted reductions of GLU and HAM muscle activity (due to changed feedback contributions) and 12% metabolic costs [158]. In the simulation study of Van den Bogert [160], *exotendons*—long elastic strings that span different leg joints—were added to an inverse model of walking. By optimizing for the most efficient arrangements of *exotendons* in the leg model, it was found that required biological joint torques and powers for generating the same walking patterns can be reduced by up to 71% and 74%, respectively. However, in real experiments, when such a passive exoskeleton was applied in conjunction with a human subject, energy expenditure of the subjects increased compared to normal walking without an exoskeleton [161].

In Malcolm *et al.* [162], a biarticular knee–ankle–foot exoskeleton with a serial arrangement of a PAM and a passive spring reduced the metabolic cost of walking more than a weight-matched monoarticular exoskeleton (to a similar level when not wearing the exoskeleton). Using an exoskeleton, metabolic reductions of up to 23% compared to walking with the unpowered system were reported in Quinlivan *et al.* [156]. Here, a multiarticular actuator simultaneously assisted hip flexion and ankle plantarflexion that reduced the biological ankle and hip torque during push-off [156]. Next to the direct energy support, it is likely that the multiarticular nature allowed for an advantageous internal energy transfer between joints [156]. The *Myosuit* aimed to assist anti-gravitational muscles at the hip and knee by a biarticular arrangement of actuators for sit-to-stand movements [157]. This system supported up to 26% and 35% of the biological hip and knee torques, respectively [157]. This shows that biarticular arrangements in an exoskeleton effectively supported propulsion [156] as well as gravity compensation [157]. Such biarticular structures might, however, be used in different leg arrangements (e.g. hip flexion and ankle plantarflexion in [156], hip and knee extension in [157], hip flexion and knee extension in [163]) and different control strategies targeting different mechanisms of assistance [164].

In rigid exoskeleton designs, biarticular actuation improved the efficiency of the exoskeleton. In the *WalkON* suit [165], the generated end-effector force per motor torque increased in some situations [166]. In another study, Zhao *et al.* [167] demonstrated that support of mono- and biarticular muscles could reduce human metabolic cost (by 10%). This was achieved by emulating muscle-like actuation at the hip and knee joint (mimicking monoarticular hip and biarticular thigh muscles) using the FMCH control method in the *LOPES II* exoskeleton [168]. However, these results should be used with caution since only two subjects participated in this pilot study [167]. Despite these studies, biarticular actuation principles were less common in rigid exoskeleton designs.

Some of the recent developments indicate potential benefits of incorporating biarticular designs in prosthetic and exoskeleton designs. However, for real-world applications and the range of subject populations, technological difficulties remain to be solved, such as human–machine interfaces, durability of hardware designs and flexible control concepts [169]. Biarticular actuation might be one useful approach to tackle some of these challenges by synchronous joint coordination and improved controllability.

## 5. Discussion

In this paper, we integrate model predictions, human experiments and robotic applications into a structured, locomotor subfunction-specific picture of biarticular muscle function. By this, we extend the conventional single joint-focused approach and advocate a generalized and more function-specific multi-joint perspective.

### 5.1. Concepts

The existence of biomechanical template models (§2.2, [1]) as observed in different animals and during different gaits and speeds [17–19] indicates a basic structure of locomotion. However, these mechanical concepts need to be reflected in the segmented human leg. Templates work with generalized coordinates (e.g. leg length and orientation) and in a low-dimensional parameter space. These generalized coordinates can be assessed by a sensible arrangement of mono- and biarticlar muscles (§2.3). This might support the control of the complex human leg (including all its DoF and muscles) in a global, simplified manner [5–7]. While such behaviour might also be generated by the neural control system in an arbitrary leg architecture, the specific arrangement of biarticular and monoarticular muscles provides a structural solution that can reduce the control effort of the motor control system [117,128,170] and make leg function more robust [171]. For a well-designed system, control can be sloppy and allow for a wider range of movements. For example, biarticular springs enlarged the stability region and robustness against spring stiffness adjustments during passive walking as predicted in the model of Dean & Kuo [14].

### 5.2. Evidence

In the presented studies in §3, biarticular muscles were found to support locomotion by a variety of features, depending on the functional requirements of the specific locomotor subfunctions stance, balance and swing.

In *stance*, muscular joint-coupling was found to synchronize neighbouring joints and distribute the energy flow along segments that enables efficient (see also review by [13]) and robust movement execution [171]. Further, almost isometric biarticular muscle operation was found, e.g. during leg extension, which resulted in more efficient torque generation than that of two monoarticular muscles. Additionally, biarticular muscles contribute to fine-tuning and proper coordination of *balance* and *swing*. For instance, multiple studies suggested a net torque based control scheme (net hip minus knee torque, extension torques defined positive) of biarticular thigh muscles [103,108] that allowed for the manipulation of GRF direction and control of angular momentum [14,41,131,172].

The multitude of biarticular features points to the idea that mono- and biarticular muscles fulfil different, subfunction-specific tasks. During *stance*, monoarticular muscles mainly power the motion. Simultaneously, by mechanically coupling adjacent joints, biarticular muscles coordinate joint movements, transfer energy and secure the zig-zag-configuration of the leg against joint overextension. In *balance* and *swing*, the contribution of mono- and biarticular muscles changes. Here, biarticular muscles power dominantly rotational motions and monoarticular muscles fine-tune the required torques since torque generation from biarticular muscles is defined by their moment arms.

In some cases, biarticular muscles also synchronize or coordinate individual locomotor subfunctions. For instance, during late stance of human walking, RF and GAS transition between *stance* and *swing* [59,98,99,108,112,173]. Moreover, GAS switches in this contribution from *balance* to *stance* when the heel lifts off the ground. When the whole foot is in contact with the ground, GAS contributes to the *balance* subfunction (by rotary forces, [39]). However, in forefoot stance, GAS supports *stance* because it can only contribute to axial forces. Future studies are needed to pinpoint the mechanisms determining the exploitation of biarticular versus coordinated monoarticular muscle strategies.

The specific function of biarticular muscles is strongly coupled to their muscle moment arms [10,62]. The overview of relevant sagittal muscle moment arms (figure 4) revealed that GAS follows the suggested moment arm ratio of 2 : 1 [10]. For biarticular thigh muscles, moment arms were greater at the hip than at the knee [62]. However, only a small number of studies investigated muscle moment arms at the hip, and study results vary due to different methods and techniques. It is therefore hard to draw clear conclusions; more research is required. For several other properties of human leg muscles, we could not find evidence for physiological differences between mono- and biarticular muscles. Only the MTC length of biarticular muscles was longer compared to monoarticular muscles. This was expected as biarticular muscles span more than one joint.

Generally, experimental studies supported predictions from conceptual template models. Even though some of these results should be used with caution due to a small number of subjects, reported evidence was very consistent across different study designs (methods) and motions (tasks). While evidence involved unperturbed tasks like standing, walking, running, cycling or load lifting, further research is needed to identify e.g. control strategies of biarticular muscles (predictive and reactive control). Here, studies on non-continuous motion tasks like gait transitions or external perturbations (like in [110]) are of particular value.

### 5.3. Applications

Several legged robots use biarticular structures to generate performant, robust or efficient motions (§4). The main motivation for this is to outsource the control effort to mechanical components (control embodiment, [128]). This is accomplished by (i) smart morphological leg arrangement and/or by (ii) facilitating intrinsic (compliant) properties that allow a certain flexibility in joint behaviour but also inherently react to external perturbations. Depending on the application, passive structures, e.g. springs or dampers, or active elements, e.g. SEA or PAM, realize biarticularity. Often, engineers use the structural compliance or muscle moment arms as design parameters to generate a desired leg behaviour [40,117,124,142,143,174]. In assistive devices, like prostheses or exoskeletons, walking economy of the wearer or efficiency of the device could be improved [155,156,162,167].

Currently, most robotic designs including industrial applications use a single actuator per DoF, instead of redundant actuation systems, in which e.g. multiple muscle-like actuators can act on a single joint. This prevents most robotic systems taking advantage of the features of biarticular actuation. However, additional actuators might also create further design challenges, like motor redundancy or undesired coupling behaviour due to fixed geometrical constraints. Both of these issues can be resolved by smart design which may be inspired from biology. In this context, abstraction and categorization as applied in this review may be useful approaches. By this, engineers can learn from template models and animals to improve artificial system designs without suffering from complexity and unwanted coupling. Some of the potentially beneficial concepts of biarticularity to be further exploited in robotic designs, are as follows:

— energy transport within a segment chain,
— improved distribution of leg inertia,
— inter-joint coordination and synchronization,
— operation of biarticular actuator with reduced power demands,
— resolving kinematic singularities.

Many of these advantages have been introduced several decades ago [12,83,84,92,94–96,104,175]. Potential implementations may enable simplified swing leg control for foot placement (as predicted in [117,153]) or reactive balance control [110]. Still, some of the features of biarticular actuators are still unexploited in robotics and await their proof-of-concept in hardware systems.

### 5.4. Conclusion

We structured this review of biarticular muscle function in two dimensions: locomotor sub-functions (*stance*, *balance* and *swing*) and methodological approaches (theoretical concepts, experimental evidence and robotic applications). Templates revealed the general organization of locomotion in different species (§2). Based on this understanding, we interpreted tangible experimental studies on biarticular muscles (§3). Finally, robotic designs (§4) transferred these mechanisms into the physical world and validated these insights and concepts. By this approach, we integrated and combined knowledge from biomechanics, biology and robotics in a unified locomotor sub-function specific perspective. For instance, the global leg function can be described by a simple leg spring [2,4]. By coordinating individual joints in the leg, biarticular muscles contribute to the generation of such global leg behaviour in human experiments [95,98] and robotic systems [131,145]. This example shows the benefit of incorporating all three domains of the research trilogy [176]: (i) conceptual modelling, (ii) human experiments, and (iii) robotic applications. Combining the expertises of biology, biomechanics and robotics seems promising to generate a deeper understanding of the structures and patterns involved in generating locomotion.

## Endnotes

1 Muscle acronyms and their location in the human leg are explained in figure 3*a*.

2 Instead of *intelligence embodiment*, introduced in Pfeifer & Bongard [129], we use the term *control embodiment* focusing on locomotion control.

3 The stiffness matrix is calculated at the end effector of a manipulator. By calculating the eigenvalues and eigenvectors of the stiffness matrix, the stiffness ellipse illustrates the compliance in different directions at the end effector. In biped robots, calculating the stiffness ellipse during stance can determine the movement direction.

4 Here, we mainly consider relevant studies since 2017. Please refer to earlier works in the review of Junius *et al.* [13].

## Data accessibility

Figures 3 and 4 contain visualizations of previously published data, references are given in the corresponding captions.
